# Study on the Shear Strength and Erosion Resistance of Sand Solidified by Enzyme-Induced Calcium Carbonate Precipitation (EICP)

**DOI:** 10.3390/ma17153642

**Published:** 2024-07-24

**Authors:** Gang Li, Qinchen Zhu, Jia Liu, Cong Liu, Jinli Zhang

**Affiliations:** 1 Shaanxi Key Laboratory of Safety and Durability of Concrete Structures, Xijing University, Xi’an 710123, China; t_bag945@126.com (G.L.); 15238796351@163.com (Q.Z.); liucong0147@outlook.com (C.L.); 2 State Key Laboratory of Coastal and Offshore Engineering, Dalian University of Technology, Dalian 116024, China; jlzhang@dlut.edu.cn

**Keywords:** EICP, standard sand, earth-rock dam, erosion resistance, shear strength

## Abstract

Sand solidification of earth-rock dams is the key to flood discharge capacity and collapse prevention of earth-rock dams. It is urgent to find an economical, environmentally friendly, and durable sand solidification technology. However, the traditional grouting reinforcement method has some problems, such as high costs, complex operations, and environmental pollution. Enzyme-induced calcium carbonate precipitation (EICP) is an anti-seepage reinforcement technology emerging in recent years with the characteristics of economy, environmental protection, and durability. The erosion resistance and shear strength of earth-rock dams solidified by EICP need further verification. In this paper, EICP-solidified standard sand is taken as the research object, and EICP-cemented standard sand is carried out by a consolidated undrained triaxial test. A two-stage pouring method is adopted to pour samples, and the effects of dry density, cementation times, standing time, and confining pressure on the shear strength of cemented standard sand are emphatically analyzed. The relationship between cohesion, internal friction angle, and CaCO_3_ formation was analyzed. After the optimal curing conditions are obtained through the triaxial shear strength test, the erosion resistance model test is carried out. The effects of erosion angle, erosion flow rate, and erosion time on the erosion resistance of EICP-solidified sand were analyzed through an erosion model test. The results of triaxial tests show that the standard sand solidified by EICP exhibits strain softening, and the peak strength increases with the increase in initial dry density, cementation times, standing time, and confining pressure. When the content of CaCO_3_ increases from 2.84 g to 12.61 g, the cohesive force and internal friction angle change to 23.13 times and 1.18 times, and the determination coefficients reach 0.93 and 0.94, respectively. Erosion model test results indicate that the EICP-solidified sand dam has good erosion resistance. As the increase in erosion angle, erosion flow rate, and erosion time, the breach of solidified samples gradually becomes larger. Due to the deep solidification of sand by EICP, the development of breaches is relatively slow. Under different erosion conditions, the solidified samples did not collapse and the dam broke. The research results have important reference value and scientific significance for the practice of sand consolidation engineering in earth-rock dams.

## 1. Introduction

The earth-rock dam is a traditional dam type with simple technology and low cost, which accounts for a large proportion of dam construction in the world. However, dam-break accidents occur frequently, and their safety has attracted much attention. There are more than 98,000 reservoirs built or under construction in China, including 756 large reservoirs, 3944 medium reservoirs, and 94,000 small reservoirs, with a total water storage capacity exceeding 900 billion cubic meters [1]. It is crucial to deal with dam failure to improve the utilization rate of resources. Traditional curtain grouting anti-seepage engineering uses cement slurry and chemical slurry, which have some shortcomings such as low permeability, high energy consumption, high costs, and environmental pollution. Therefore, it is an important task and necessary measure for geotechnical engineering to develop energy-saving, emission-reducing, eco-environmental, economical, and efficient dam seepage control and reinforcement technology. Plant-derived enzyme-induced calcium carbonate precipitation technology is a new solidification method. The mineralization principle is that urease is extracted from plants, and urea is decomposed into ammonium ions and carbonate ions under the catalytic action of urease. Subsequently, carbonate ions react with the calcium source to form a CaCO_3_ precipitate, which bonds the soil particles to form a whole, achieving the purpose of solidification [2,3,4,5,6,7]. The EICP technology has the characteristics of high efficiency [8], environmental friendliness [9], economic benefit [10], durability [11], etc. This method not only has many applications in reinforcement but also involves the restoration and protection of ancient buildings [12], replacing some civil engineering materials [13], improving the thermal conductivity of backfill materials and soil [14], etc.

Under normal circumstances, the shear strength of soil is an important mechanical index of soil that is closely related to the bearing capacity of the foundation, the earth pressure of the retaining wall, and the stability of the slope. Alwalan et al. [15] conducted direct shear tests on consolidated sand spraying through EICP technology in four different ways: namely spraying, mix and compact, percolation, and injection. In the spraying method, EICP solution is directly sprayed on the top of the sample through a plastic spray bottle and then penetrates the sample. Compared with the untreated sample, the peak shear strength of sand increased by about 2.3 times. Meng et al. [16] found that the amount of calcium carbonate generated in the multiple-phase method is at least four times higher compared to the one-phase method. After 20 applications of cementation solution, the unconfined compressive strength of the EICP-treated sand exceeds 10 MPa, with a CaCO_3_ content of 20%. He et al. [17] discovered that after EICP treatment of soil slopes was carried out four times in dry and humid environments, the permeability resistance increased to 92.1 N and 71.5 N in slope runoff erosion experiments, respectively, which were 7.7 times and 11.3 times higher than those in one treatment round. Wang et al. [18] found that there is a relationship between the cement solution concentration and the unconfined compressive strength of the EICP-treated sand soil. When grouting times are N = 4, as the cement solution concentration increases from 0.75 mol/L to 1.5 mol/L, the unconfined compressive strength continued to increase, reaching a maximum of 9.87 MPa, which is an improvement of 1.87 times. Gao et al. [19] found that soybeans crude extract-induced calcium carbonate precipitation (SICP) improved the strength of sandy soil. The experiment showed that the surface strength of SICP-treated sandy reached 306.2 kPa compared with untreated sandy soil, an increase of 1813.75%. Almajed et al. [20] found that compared to untreated samples, the samples treated with the EICP and sodium alginate (SA) combination had the lowest erosion rate. SA of 0.5% biopolymer and EICP solution significantly enhances the surface strength and the amount of carbon precipitation in sandy soil. Wu et al. [21] found that when the urease activity increased from 2.95 U/mL to 5.39 U/mL, the cementing solution increased from 0.25 M to 0.75 M, and the unconfined compressive strength of the EICP-solidified sand was enhanced from 180.58 kPa to 1850.70 kPa, which improved 9.25 times. Alotaibi et al. [22] found that in a life cycle assessment (LCA) experience, the abiotic depletion potential of EICP-treated sand in terms of capacity was reduced by nearly 90% compared with portland cement (PC), and the global warming potential of soil capacity was 3% lower than that of PC. Miao et al. [23] included that the EICP has higher production efficiency and production in a wide temperature range (10 ± 70 °C), significantly improving the water retention performance of the material, which is more suitable for desert environments. It is included that with the increase in spraying times, the urease mineralization method can better resist wind. Lee et al. [24] found that in EICP-solidified soil, when the soybean solution was 140 g/L and the urea–CaCl_2_ solution was 3 M, the unconfined compressive strength increased from 273 kPa to 870 kPa when curing time was 7 days and 28 days, respectively. Baruah et al. [25] found that the influence of urea, calcium chloride, and urease enzyme on the development of sand strength was studied using unconfined compressive strength. A combination of 0.5 M urea, 1 M CaCl_2_, and 6 g/L urease enzyme was used as the bonding medium to obtain the maximum calcium carbonate precipitation. Xu et al. [26] obtained that the optimal extraction time and dosage of urease solution in the EICP-treated sand cushion layer were 1 h and 100 g/L. As the number of EICP treatments increases, the content of calcium carbonate increases and the highest dynamic deformation modulus (Evd) reaches 50.55 MPa. Shen et al. [27] discovered that the addition of basal fiber (BF) and polyvinyl acetate emulsion (PVAC) in the EICP experiment resulted in high surface strength (SS) with the addition of 50 g/L PVAC. After treatment with EICP-0.5% BF, EICP-30 g/L PVAC, and EICP-50 g/L PVAC, the sandy soil exhibits high erosivity.

The mechanical properties of EICP-solidified sand have improved significantly, but the erosion resistance still needs to be studied. Through the triaxial shear strength test, the influence factors of dry density, cementation times, standing time, and confining pressure on the strength of solidified sand are discussed. The correlation between cohesion, internal friction angle, and CaCO_3_ precipitation amount is established, and the optimum conditions for solidifying standard sand by EICP are determined. At the same time, the effects of erosion angle, flow rate, and time on erosion performance were analyzed by an erosion model test, and its durability under erosion was evaluated. These achievements provide an important reference for reinforcement and seepage control of earth-rock dams.

## 2. Materials and Methods

### 2.1. Test Materials

**Figure 1:**
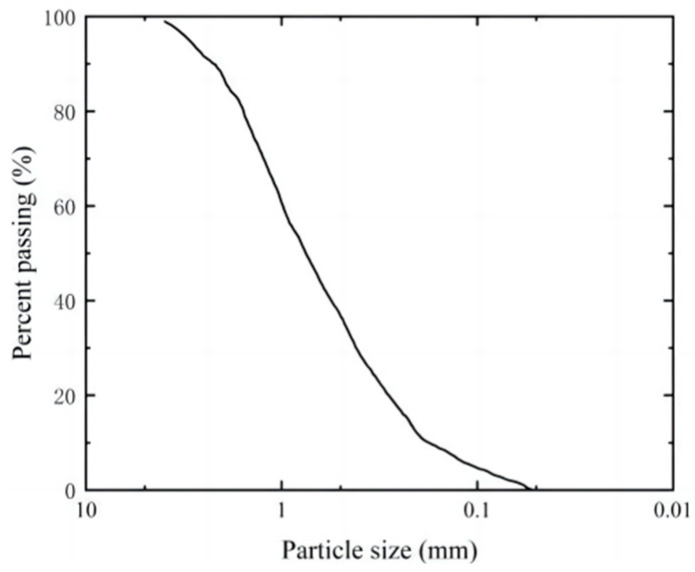
Sand particle size distribution curve.

The test adopts (GB/T 17671-1999) [28] standard sand from Xiamen, China, and the parameters are shown in Table 1 below. According to its nonuniformity coefficient (*C*_u_) and curvature coefficient (*C*_c_), the sand is judged to be fine sand with poor particle gradation. According to the standard for geotechnical test methods (GB/T 50123-2019) [29], the sand particle size distribution curve is shown in Figure 1. Soybeans were purchased in the market, and their origin was Suihua City, Heilongjiang Province. The reagents used in the test are urea (Tianjin Hengxing Chemical Reagent Manufacturing Company, Tianjin, China) and calcium chloride (Suzhou Wuzhong District Luzhi Sheng Da Drying Reagent Company, Suzhou, China), and the purity is more than 99.0%.

### 2.2. Sample Preparation

**Figure 2:**
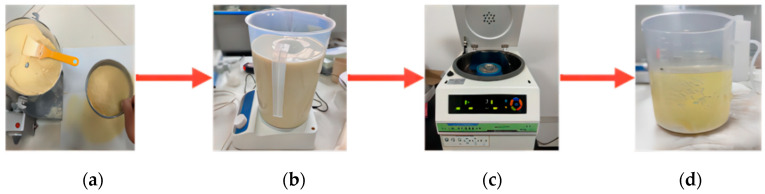
Separation and extraction process of soybean urease: (**a**) crushed; (**b**) stirring sample powder; (**c**) centrifugation of sample; (**d**) urease.

The size of the standard sand sample is 39.1 mm in diameter and 80 mm in height. To carry out the triaxial shear strength test, a PVC pipe with an inner diameter of 39.1 mm and a height of 150 mm was selected as the die. The test steps should strictly follow the standard for geotechnical test methods (GB/T 50123-2019) [29]. Samples are made in four layers, and shaving treatment is needed between each layer. Before loading, lay three layers of filter paper on the bottom of the sample, and after loading, lay another layer of filter paper on the top. The bottom of the mold shall be plugged with a rubber plug with holes, and the reserved holes shall be connected to the liquid outlet. Urease is extracted from soybeans, urease activity is measured in a conductivity meter and the steps of extracting urease solution include the following: crushing dried soybean until the particle size is less than 0.10 mm, weighing an appropriate amount of soybean flour and adding it to deionized water to achieve a concentration of 100 g/L, stirring the solution for 30 min, and then refrigerating for 24 h at a temperature of 4 °C. The solution was centrifuged at high speed for 15 min, and the supernatant was taken to obtain the urease solution. The concentration of urea and calcium chloride solution was adjusted to 1.25 mol/L, and the pH value was 8.0. The sample was perfused by a two-stage method: first, 20 mL of urease solution, then 10 of mL calcium chloride solution, and 10 mL of urea solution, with an interval of 24 h each time. After completion, we rinsed with purified water three times to stop the reaction. Finally, the sample is saturated in an air-pumping vacuum saturation cylinder. Separation and extraction of soybean urease are shown in Figure 2.

### 2.3. Test Methods

According to the standard for geotechnical test methods (GB/T 50123-2019) [29], the triaxial shear strength test adopts the KTL-LDF 50 soil static triaxial instrument (Xi’an Kangtuoli Instrument and Equipment Co., Ltd., Xi’an, China) for the consolidated undrained test. During the test, the loading mode was set to be controlled by a strain rate of 0.10%/min, and the test was stopped when the axial strain reached 15%. Based on relevant research [30,31,32,33], dry density, cementation times, standing times, and confining pressure were determined, and the test scheme is shown in Table 2. Carry out an erosion model test, analyze the influence of erosion angle, erosion flow rate, and erosion time on erosion resistance, and verify the erosion resistance of EICP-cemented sand. See Table 3 for the test scheme.

**Figure 3:**
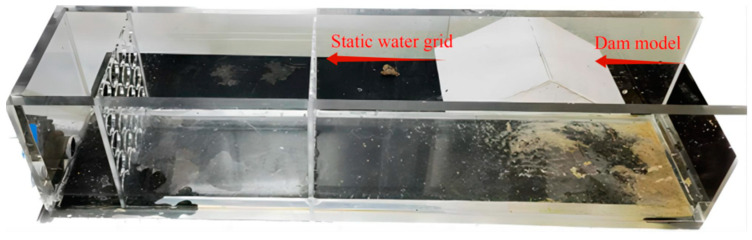
Erosion resistance model test chamber.

Figure 3 shows a model box for erosion resistance in which plain sand is presented in the form of natural accumulation with a particle density of 1.65 g/cm^3^, which is used for the erosion model test. The size of the dam model is 160 mm in length and 100 mm in width. This kind of experiment was carried out in a model box with a length of 50 cm, a width of 10 cm, and a depth of 12 cm. As shown in Figure 3, a self-circulating water pump is used for water supply, the dam model is placed at a position 2/3 away from the water inlet to prevent the uneven erosion of water flow, and a static water grid is set at a position 1/3 away from the water inlet. During the dam cementation process, urease solution (100 g/L), calcium chloride solution (1.25 mol/L), and urea solution (1.25 mol/L) were uniformly sprayed on the surface of the dam sample using T_70103405A adjustable spray cleaning bottle (Thermo Fisher Scientific, Massachusetts, USA). To ensure that the solidified dam samples have good shaping performance, a small amount of deionized water was added for mixing before spraying the solidification liquid.

## 3. Results and Discussion

### 3.1. Analysis of the Influence of Dry Density on the Strength of Solidified Sand

**Figure 4:**
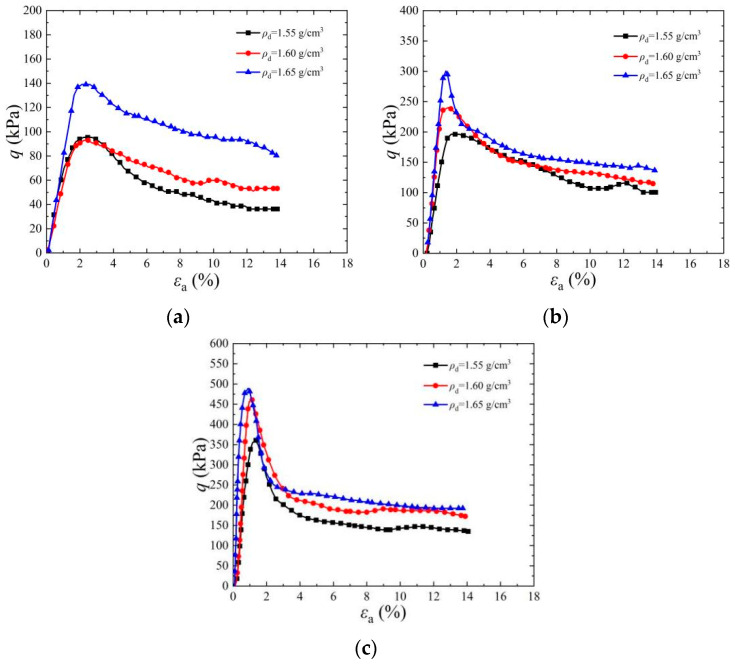
Effect of dry density on stress–strain curve of EICP-cemented standard sand: (**a**) *t*_1_ = 1 d, *n* = 2; (**b**) *t*_1_ = 1 d, *n* = 4; (**c**) *t*_1_ = 1 d, *n* = 6.

Figure 4 shows a stress–strain diagram of a cemented specimen under a confining pressure of 50 kPa. It can be seen from the figure that under the same confining pressure, with an increase in initial dry density, the peak strength of deviator stress increases with different standing times and cementation times. When the cementation times increased from two to six times and the dry density increased from *ρ*_d_ = 1.55 g/cm^3^ to *ρ*_d_ = 1.65 g/cm^3^, the peak strength of deviator stress increased significantly and the corresponding axial strain decreased gradually. The peak strength of cemented specimens is 518.39 kPa, 520.3 kPa, and 569.72 kPa when the dry density is 1.55 g/cm^3^, 1.60 g/cm^3^, and 1.65 g/cm^3^, respectively, when the cementation times are six times, the confining pressure is 50 kPa, and the standing time is 1 d. The main reason for the above phenomenon is that during the EICP solidification process of sandy soil, urease accelerates the urea reaction and combines with the calcium source to produce CaCO_3_ [2]. The produced CaCO_3_ crystals mainly cement loose sand particles into a whole through bonding and filling. With the increase in sample dry density, the pore size between sand particles decreases, which improves the cementation efficiency of CaCO_3_ and then makes the peak strength of the sample increase accordingly [34]. Under confining pressure, the cohesion and internal friction angle between sand particles increase, which makes the shear strength of cemented samples improve.

### 3.2. Analysis of the Influence of Cementation Times on the Strength of Solidified Sand

**Figure 5:**
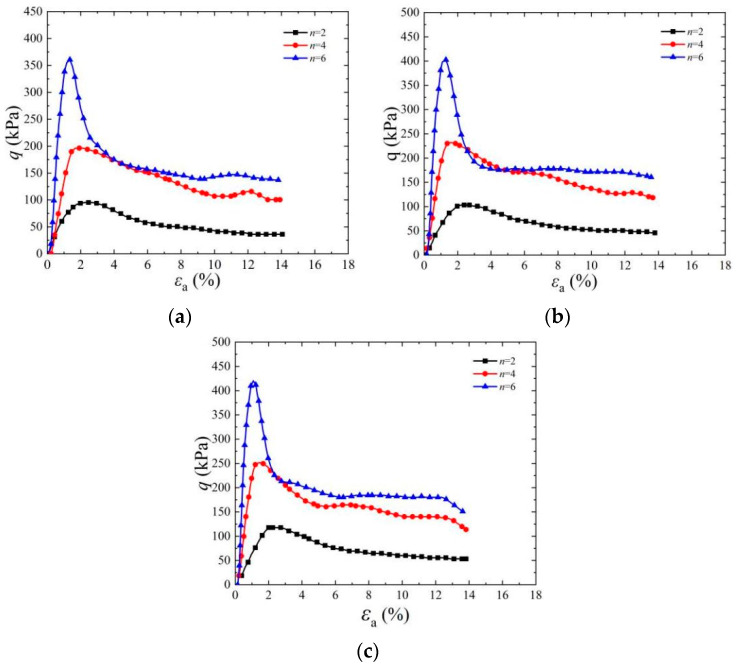
Effect of cementation times on stress–strain curve of EICP-cemented standard sand (*σ*_3_ = 25 kPa): (**a**) *ρ*_d_ = 1.65 g/cm^3^, *t*_1_ = 1 d; (**b**) *ρ*_d_ = 1.65 g/cm^3^, *t*_1_ = 3 d; (**c**) *ρ*_d_ = 1.65 g/cm^3^, *t*_1_ = 5 d.

Figure 5 shows the stress–strain diagram of the cemented specimen under a confining pressure of 25 kPa. It can be seen from the figure that under the same confining pressure, with the increase in cementation times, the amount of CaCO_3_ generated gradually increases and the peak intensity of deviator stress increases continuously. When *ρ*_d_ is 1.65 g/cm^3^, the confining pressure is 25 kPa, the standing time is 5 d, and cementation times are two, four, and six, respectively, the corresponding deviator stress peaks are 186.06 kPa, 385.43 kPa, and 548.10 kPa, respectively. The axial strain corresponding to the peak value of deviator stress is 1.85~2.51% when the cementation times are two times, and decreases to 0.78~1.84% when the cementation times increase to four times and six times. The main reason for the above phenomenon is that with the increase in cementation times from 2 times to 6 times, CaCO_3_ crystals produced by the reaction between urease and cementing solution are also increasing, so the consolidation strength of samples is also increasing. In cemented specimens, when the deviator stress reaches its peak value, the brittle failure phenomenon will appear. At this time, the deviator stress decreases rapidly, and the more cementation times, the more obvious the brittle failure characteristics are, and this is consistent with the findings of reference [35]. Consequently, the axial strain of cemented specimens decreases accordingly.

### 3.3. Analysis of the Influence of Standing Time on the Strength of Solidified Sand

**Figure 6:**
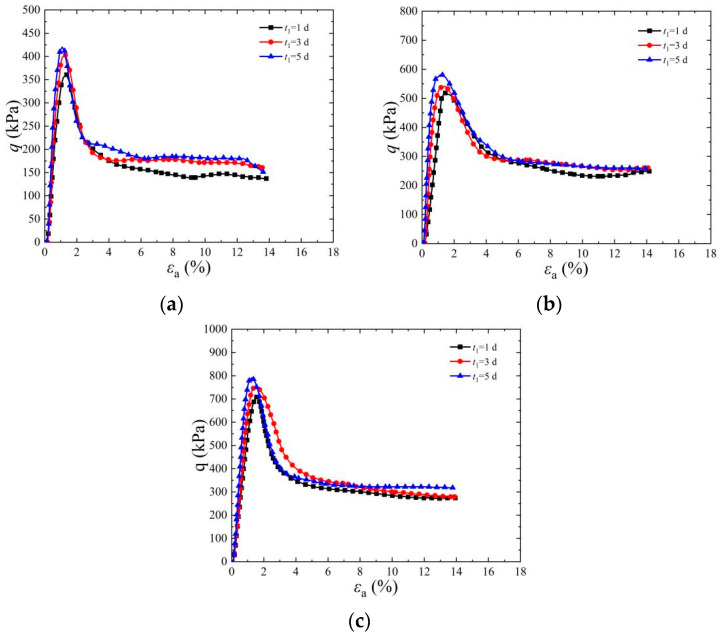
Effect of standing time on stress–strain curve of EICP–cemented standard sand (*n* = 6): (**a**) *ρ*_d_ = 1.65 g/cm^3^, *σ*_3_ = 25 kPa; (**b**) *ρ*_d_ = 1.65 g/cm^3^, *σ*_3_ = 50 kPa; (**c**) *ρ*_d_ = 1.65 g/cm^3^, *σ*_3_ = 100 kPa.

Figure 6 is a stress–strain diagram of a cemented specimen at a dry density of 6. It can be seen from the figure that, under the same dry density, the peak strength of deviator stress of cemented specimens increases with the increase in confining pressure and standing time. When *ρ*_d_ is 1.65 g/cm^3^, the confining pressure is 100 kPa, cementation times are 6, standing time is 5 days, and the maximum deviator stress of the specimen reaches 988.20 kPa. Compared with the peak strength of deviator stress under the same condition, when the standing time is 1 d, it increases by 12.52%. During the solidification process of EICP, urease catalyzes the production of carbonate ions from urea. The content of CaCO_3_ precipitate produced by the combination of carbonate ions and calcium source ions has a significant impact on the strength of the sample [3]. This phenomenon is mainly due to the continuous reaction between the urease solution and cementing solution in the sample with the passage of time. The longer the standing time, the more sufficient the curing reaction, and then more CaCO_3_ precipitates are generated from cement sand particles, thus increasing the peak value of the deviator stress of the sample [36]. The test results show that when the standing time is 5 days, the deviator stress of the cemented specimen reaches its highest value. However, when the standing time is 1 day, the peak value of deviator stress is low due to the insufficient reaction between urease and cementing solution.

### 3.4. Influence of Confining Pressure on Strength of Solidified Sand

**Figure 7:**
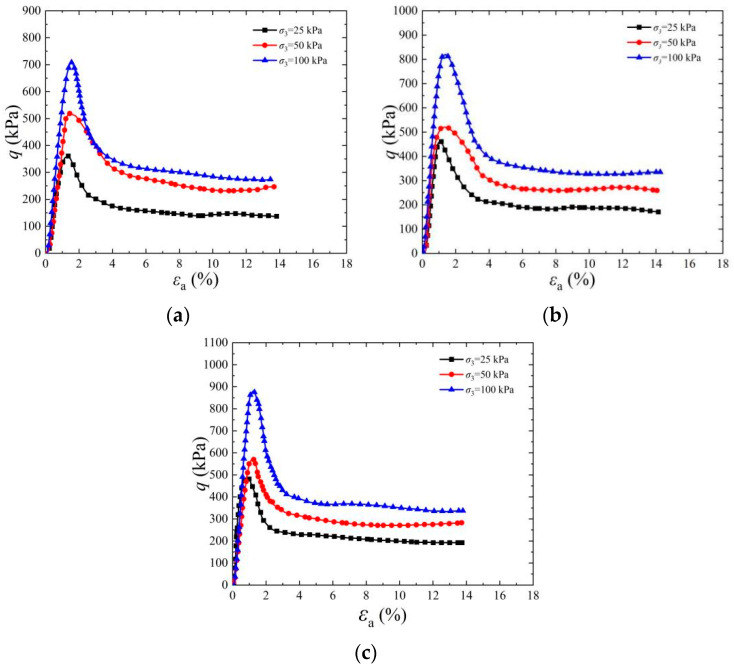
Effect of confining pressure on stress–strain curve of EICP-cemented standard sand (*n* = 6): (**a**) *ρ*_d_ = 1.55 g/cm^3^, *t*_1_ = 1 d; (**b**) *ρ*_d_ = 1.60 g/cm^3^, *t*_1_ = 1 d; (**c**) *ρ*_d_ = 1.65 g/cm^3^, *t*_1_ = 1 d.

Figure 7 shows the stress–strain diagram of cemented specimens under different confining pressures. With an increase in the in situ depth of soil, its confining pressure increases accordingly. The figure shows the influence of different confining pressures on the shear strength characteristics of EICP-solidified specimens. It can be seen from the figure that when the cementation times are six, the residual strength of cemented samples under low confining pressure is significantly lower than the peak strength, and the stress–strain curve shows an obvious strain softening phenomenon. The peak strength of the cemented specimens at 25 kPa, 50 kPa, and 100 kPa is 482.12 kPa, 569.92 kPa, and 880.04 kPa, respectively, when the cementation times are 6 times and the standing time is 1 d. The main reason for the above phenomenon is that with the increase in confining pressure, the closer the contact between CaCO_3_ crystal and soil particles, the more contact points there are, which leads to the enhancement of adhesion and friction between CaCO_3_ crystal and soil particles, and this is consistent with the findings of reference [37]. When the specimen is subjected to an external load, the force conversion and transfer effect occur between the CaCO_3_ crystal and soil particles, and they will share the external load, thus further improving the shear strength of EICP-solidified standard sand.

### 3.5. Failure Mode Analysis of Samples

**Figure 8:**
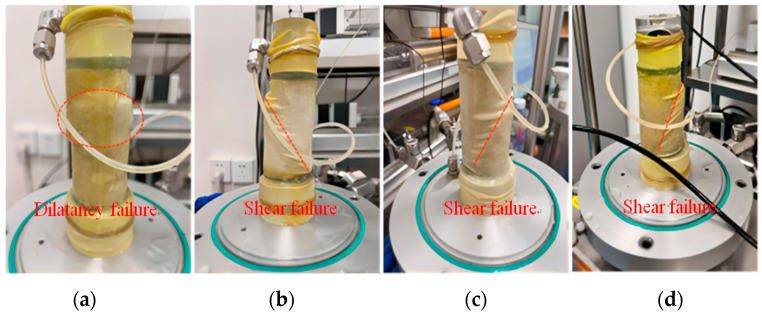
Failure modes of EICP-cemented specimens under different cementation times and different confining pressures: (**a**) *n* = 2, *σ*_3_ = 25 kPa; (**b**) *n* = 4, *σ*_3_ = 50 kPa; (**c**) *n* = 6, *σ*_3_ = 25 kPa; (**d**) *n* = 6, *σ*_3_ = 100 kPa.

Figure 8 shows the failure modes of EICP-cemented samples under different cementation times and confining pressures. As shown in Figure 8a, when the cementation times are 2, with the continuous increase in strain, the specimen state first appears as shear shrinkage and then dilatancy failure, forming an indistinct shear band. With the increase in strain, the shear band becomes clear gradually, and the specimen is destroyed at the shear band. As shown in Figure 8b, the shear phenomenon is basically consistent with that in Figure 8a, but with the increase in cementation times, the surface strength of the sample is significantly improved compared with that in Figure 8a. However, with the continuous increase in confining pressure times, the surface shear failure phenomenon is gradually aggravated, and the shear band is becoming more and more obvious. As shown in Figure 8c, the shear phenomenon is basically consistent with Figure 8a, and the shear failure of the specimen decreases with the increase in cementation times. In Figure 8d, the continuous strain growth rate is faster than that in Figure 8a. At this time, the phenomenon is not local shear failure, but the cementation between sand particles and CaCO_3_ is destroyed first. With the increase in cementation times and confining pressure, the time of shear back formation is relatively early, and the degree of shear failure is more thorough. Experiments indicate that local deformation occurs earlier at low confining pressure than at high confining pressure. Strain softening and obvious shear bands are formed in the specimens, and then the strength decreases obviously. This shows that EICP-solidified standard sand has poor plasticity under low confining pressure and a high cementation degree and is more prone to brittle failure, and this is consistent with the findings in [38].

### 3.6. Analysis of the Relation between Cohesion, Internal Friction Angle, and CaCO_3_ Formation

**Figure 9:**
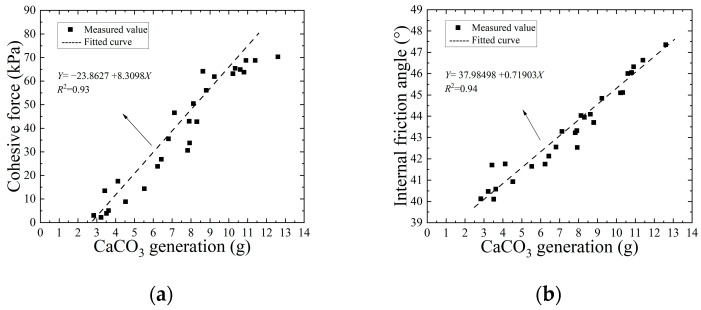
Relationship between cohesion and internal friction angle and calcium carbonate production: (**a**) cohesion; (**b**) angle of internal friction.

Figure 9 is the regression analysis curve of cohesion, internal friction angle, and CaCO_3_ formation of the EICP-solidified standard sand sample. After EICP treatment of standard sand samples, it was found that the increase in cohesion brought about an improvement in shear strength. As shown in the figure, when the content of CaCO_3_ increases from 2.84 g to 12.61 g, the cohesive force and internal friction angle change to 23.13 times and 1.18 times, respectively. It can be seen that there is a positive correlation between them, and the determination coefficient reaches 0.93 and 0.94, respectively, indicating that there is a significant correlation between cohesion and CaCO_3_ production. The main reason for this phenomenon is that CaCO_3_ crystals formed during the EICP process can fill and cement sand particles and enhance the adhesion and friction between sand particles. Therefore, the loose sand particles can be cemented into a whole and bear the external load together, thus improving its shear strength. Therefore, there is a significant positive correlation between the amount of CaCO_3_ produced and the cohesion and internal friction angle of the specimen, so the main way to improve the shear strength of standard sand using EICP technology is to increase its cohesion.

## 4. Analysis of Erosion Characteristics of EICP-Solidified Sand

### 4.1. Erosion Performance Analysis of Uncemented Dam

**Figure 10:**
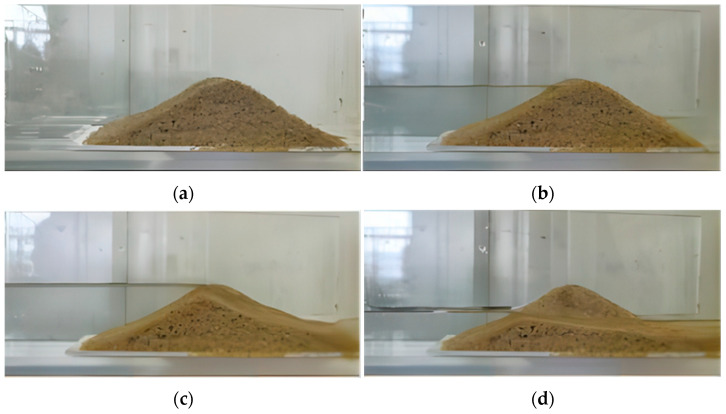
Model test of standard sand dam: (**a**) uncemented specimen; (**b**) the water flow reaches the dam crest; (**c**) water erosion; (**d**) end of erosion.

Figure 10 is a standard sand dam with an uncemented α = 30° and *Q* = 1 L/min, and the erosion resistance model test is carried out. As shown in the figure, when the water level overflows the height of the dam crest, a narrow breach at the dam crest can be observed immediately, and the breach will spread and expand rapidly to both sides [39]. The erosion process is mainly affected by water flow, and the continuous expansion of the breach will also cause the collapse of the soil on both sides. The eroded particulate matter will be transported to the downstream slope toe for deposition along with the water flow, which leads to the slope of the slope toe gradually slowing down until it reaches a certain stable state. The whole erosion process is very short, lasting about 6 s, and its failure speed is extremely rapid, which cannot be directly compared with the dam samples after EICP cementation treatment. When the water flows over the top of the embankment, it will cause erosion on the surface of the embankment and produce local shear stress. Once this shear stress reaches or exceeds its critical value, it will lead to erosion failure, which will scour the soil particles to the foot of the slope and deposit them. The breach usually occurs in the weak link of the dam because the shear stress at the breach is higher than in the surrounding area, so the breach will expand rapidly.

### 4.2. Erosion Angle Analysis

**Figure 11:**
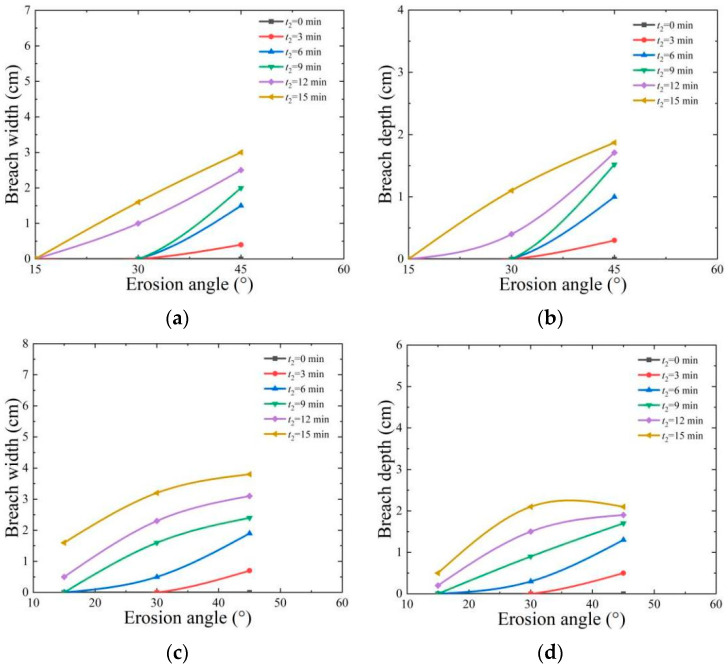
Influence of erosion angle on the top depth and widening of dam central axis breach: (**a**) *Q* = 5 L/min, breach width; (**b**) *Q* = 5 L/min, breach depth; (**c**) *Q* = 7 L/min, breach width; (**d**) *Q* = 7 L/min, breach depth.

Figure 11 shows the relationship between erosion angle, breach width, and breach depth under erosion flow rates (*Q*) of 5 L/min and 7 L/min. It can be seen from the figure that within 3 min, the depth of breach changes little in the erosion test, and the dam is relatively stable. When the erosion flow rate is 33 L/min and the erosion angles are 15°, 30°, and 45°, there is no breach in the dam specimen. As the angle increases to 30° and 45°, the breach appears, and the depth and width of the breach gradually expand with the passage of erosion time. The reason for the above phenomenon is that on the surface of the dam treated with EICP, urease accelerates the combination of carbonate ions and calcium ions to form CaCO_3_ crystal shells [5]. The EICP test shows that the smaller the erosion angle, the lower the shear stress caused by overtopping erosion, the higher the dam stability, and the longer the dam break time. However, the larger the erosion angle is, the higher the top shear stress of water flow [40], and with the increase in erosion time, the breach will appear in the fragile part of surface cementation, but the breach develops relatively slowly due to the larger cementation depth.

### 4.3. Erosion Flow Analysis

**Figure 12:**
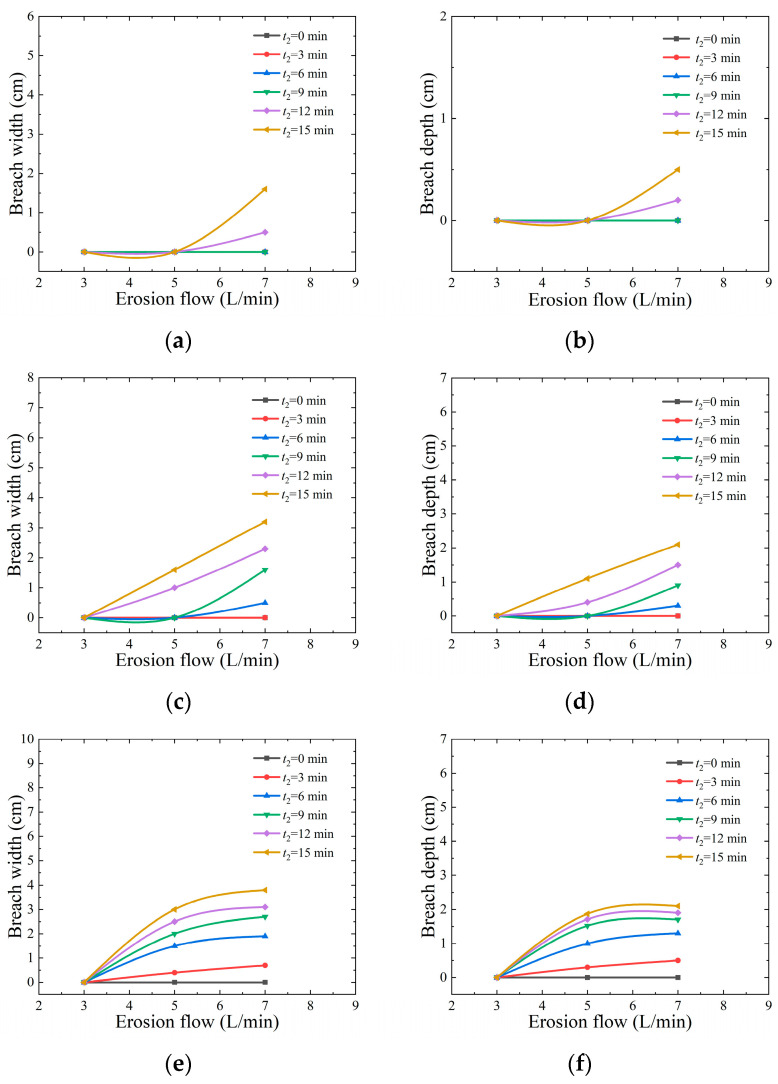
Influence of erosion discharge on the top depth and widening of dam central axis breach: (**a**) *α* = 15°, breach width; (**b**) *α* = 15°, breach depth; (**c**) *α* = 30°, breach width; (**d**) *α* = 30°, breach depth; (**e**) *α* = 45°, breach width; (**f**) *α* = 45°, breach depth.

Figure 12 shows the curves of erosion flow rate (*Q*) versus the breadth and depth of the breach at 15, 30, and 45 erosion angles. It can be seen from the figure that no obvious breach phenomenon is observed when the erosion angle is 15 and the erosion flow rate is 3 L/min. However, when the erosion flow rate is increased to 7 L/min, a significant breach phenomenon appears. The experimental results show that as the increase in erosion angle, the breadth and depth of the breach show an obvious increasing trend. At the same time, with the continuous increase in erosion flow, the width and depth of the breach are gradually expanded, which is consistent with the findings of reference [41]. The appearance of this phenomenon is due to the corresponding enhancement of water impact force caused by the increase in erosion flow rate, which in turn increases the shear stress for overtopping failure. Once the dam breaks, its energy release will be more intense. If the water flow rate continues to increase, the time of rapid erosion will be further shortened in the process of overtopping erosion. This will accelerate the development of breaches and the scale of the secondary collapse, which will lead to an increase in breach flow.

### 4.4. Erosion Time Analysis

**Figure 13:**
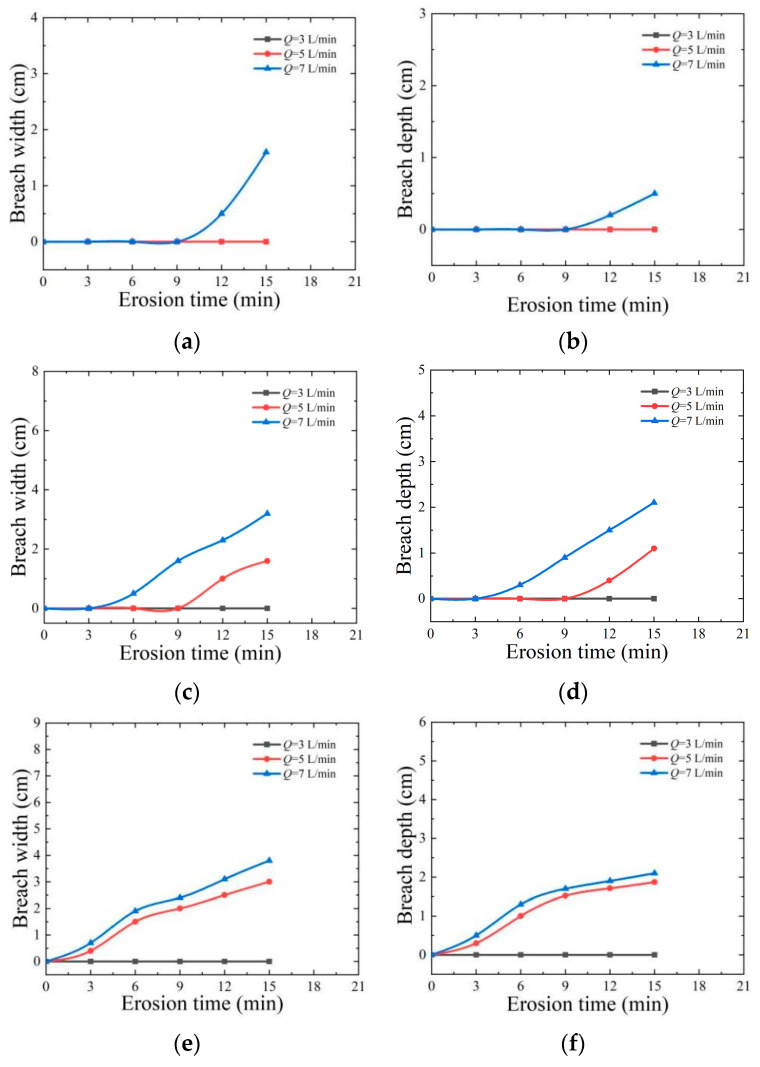
Influence of erosion time on the top depth and widening of dam central axis breach: (**a**) *α* = 15°, breach width; (**b**) *α* = 15°, breach depth; (**c**) *α* = 30°, breach width; (**d**) *α* = 30°, breach depth; (**e**) *α* = 45°, breach width; (**f**) *α* = 45°, breach depth.

Figure 13 shows the relationship between erosion time, breach width, and breach depth at 15, 30, and 45 erosion angles. It can be seen from the figure that after the dam breach occurs, the depth and width of the breach will gradually expand with the passage of erosion time. When the erosion flow rate is 3 L/min and the erosion angle is 15°, there is no breach in the dam specimen. However, with the increase in erosion angle and flow rate, the expansion speed of the breach will accelerate at first and then tend to be stable. The reason for this phenomenon is that the overtopping dam failure belongs to the type of scouring failure, which first occurs at the contact between the dam crest and the downstream slope. In the case of a small flow rate, the scouring speed is relatively slow [41]. During the EICP mineralization process, urease-catalyzed hydrolysis of urea produces carbonate ions [6]. The calcium carbonate precipitate produced by carbonate ions and calcium ions tightly binds with the sand particles on the surface of the dam, forming a strong hard shell that prevents the shear stress generated by water flow from damaging the top and downstream of the dam. However, with the increase in water flow rate and erosion angle, the shear stress will increase accordingly. Therefore, under the action of erosion time, the breach first appeared at the weak surface cementation site, where the shear stress was higher than the surrounding area, and the breach developed rapidly. However, due to the large cementation depth of EICP, the development speed of the breach is relatively slow.

### 4.5. Analysis of Erosion Failure Mode

**Figure 14:**
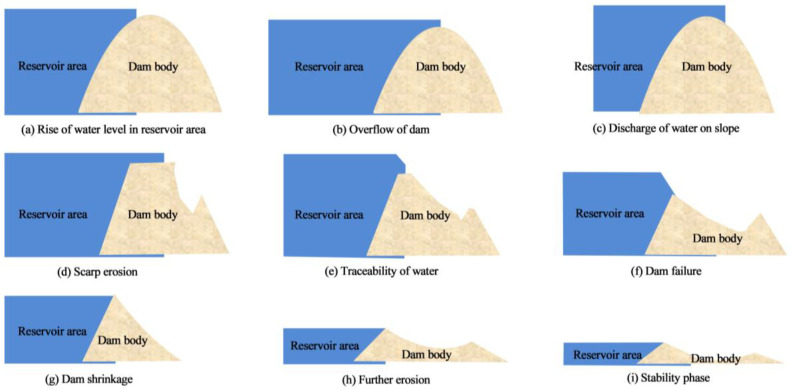
Failure process of dam overtopping: (**a**) rise of water level in reservoir area; (**b**) overflow of dam; (**c**) discharge of water on slope; (**d**) scarp erosion; (**e**) traceability of water; (**f**) dam failure; (**g**) dam shrinkage; (**h**) further erosion; (**i**) stability phase.

Figure 14 shows the failure process of dam overtopping. Overtopping dam failure is a form of traceability scouring, and dams without EICP reinforcement will collapse and break under current erosion. The failure begins in the contact area between the dam crest and the downstream slope, during which the current erosion forms a gully and extends upstream. As time goes by, the gully expands and deepens to form an obvious breach. When the breach extends to the upstream edge of the dam body, the flow rate increases sharply and the erosion intensifies, which leads to the expansion and deepening of the breach and the collapse of the dam bodies on both sides. After the breach runs through, the water flow velocity increases sharply, the water level drops sharply, the dam erosion becomes more intense, the breach continues to expand and deepen, and the collapse of the dams on both sides intensifies. Due to the uneven particle distribution and structure in the dam, the breach is often asymmetric, which leads to the flow around the breach and aggravates the erosion at the foot of the slope. With the continuous discharge of water flow, the upstream water level gradually decreases, and the erosion of the upstream part weakens, finally emerging from the water surface. At this time, the main erosion focus of the breach shifts to the downstream side wall, and the water flow continues to erode the foot of the breach slope, causing the slope to collapse [42].

**Figure 15:**
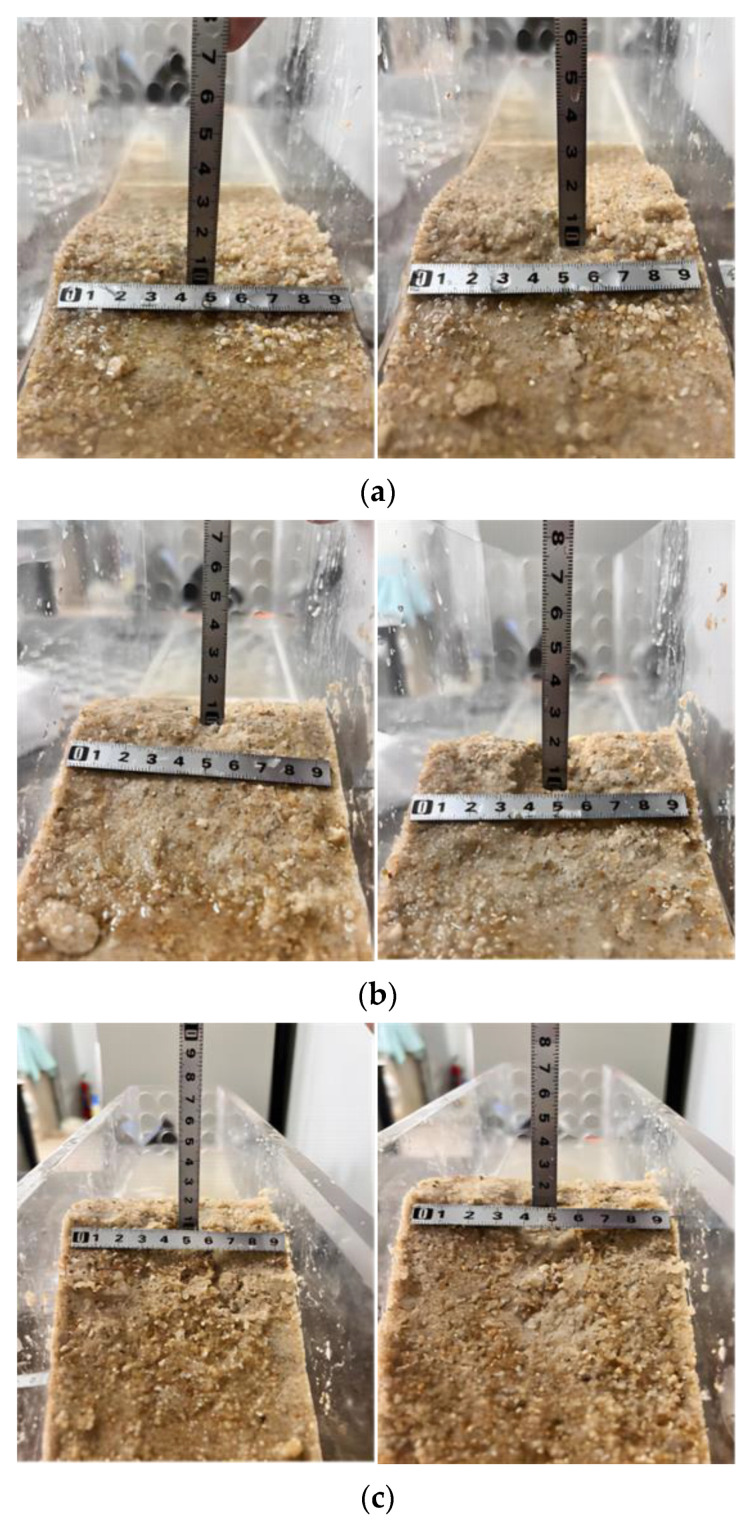
Erosion failure mode: (**a**) *α* = 15°; (**b**) *α* = 30°; (**c**) *α* = 45°.

Figure 15 shows the erosion pattern of the dam strengthened by EICP after failure. As shown in the figure, there is no serious collapse or dam break under various erosion conditions. When erosion occurs, the breach of the sample is uneven in shape. Weak cementation will wash the massive particles to the foot of the slope under the erosion of water flow. As shown in Figure 15a, when the erosion angle is 15, the breach is mostly shallow and wide. As shown in Figure 15a–c, when the erosion angles are 30 and 45, the breach is mostly deep and thin. The main reason for the above phenomenon is that after EICP treatment, CaCO_3_ crystals cover the surface of sand particles, which can wrap and cement loose sand particles into a whole, and the friction and adhesion between sand particles increase at the same time, which is consistent with the findings of reference [43]. Compared with uncemented dam samples, the anti-erosion ability is significantly improved and the integrity is stronger when subjected to water erosion. At the same time, due to the deep cementation, the weak part of the cementation is washed to the foot of the slope by water flow after the breach, while the remaining part can still resist the erosion of water flow.

## 5. Conclusions

The effects of dry density, cementation times, standing time, and confining pressure on the shear strength of EICP-solidified Aeolian sand were analyzed through a triaxial consolidated undrained shear test. The quantitative relationship between CaCO_3_ generation, cohesion, and internal friction angle was established. The effects of erosion angle, erosion flow rate, and erosion time on the erosion resistance of EICP-solidified sand were analyzed through the erosion model test. The main conclusions are as follows:

1. When the dry density of the sample is high, the smaller the distance between sand particles, the better the cementation effect of CaCO_3_. With the increase in the cementation times, the strength of standard sand increases, and the brittle failure is obvious. With the increase in standing time, the peak strength of the deviator stress of standard sand also increases.
2. When the sample with low CaCO_3_ content reaches its peak strength, the internal structure changes little, the difference between residual strength and peak strength is small, and the strain softening is not obvious. When the sample with high CaCO_3_ content reaches peak strength, the internal CaCO_3_ crystal is destroyed, the integrity of sand particles is damaged, the shear strength is greatly reduced, the difference between residual strength and peak strength is large, and the strain softening is obvious.
3. The local deformation of the specimen under low confining pressure is earlier than that under high confining pressure. Under low confining pressure and high cementation, the deviator stress increases rapidly with the increase in strain, then decreases or stabilizes after the peak value, and the specimen appears to have strain-softening characteristics, with the peak value appearing as a shear band. The strength of EICP-solidified sand is obviously decreased, which indicates that EICP-solidified sand has poor plastic deformation ability under low confining pressure and high cementation levels and is more prone to brittle failure.
4. With the increase in CaCO_3_ content, the cohesion and the angle of internal friction increase, showing a positive correlation. CaCO_3_ crystals produced in the EICP process play a role in filling and cementing sand particles, which increases the cohesion and internal friction angle between sand particles and makes the loose sand particles be cemented together to bear the external load together, thus significantly improving their shear strength.
5. The smaller the erosion angle, the better the stability of the dam, and the longer the dam break time. When the erosion angle is 15° and the erosion flow rate is 3 L/min, there is no obvious damage, and the overall integrity of the dam sample is good. The larger the erosion angle, the greater the overtopping shear stress of the water flow. With the increase in erosion time, the breach appears at the weak surface cementation.
6. With the increase in erosion flow, the impact capacity of water flow is gradually enhanced. When overtopping failure occurs, the shear stress will increase, and at the same time, when the dam breaks, the released energy will become greater, which will lead to an increase in the degree of harm caused by it. In addition, in the process of overflow scouring, the increase in flow rate will accelerate the scouring speed and make the breach gradually larger.

## Figures and Tables

**Table 1:** Xiamen ISO standard sand parameters.

*ρ*_dmax_ (g/cm^3^)	*ρ*_dmin_ (g/cm^3^)	*d*_10_ (mm)	*d*_30_ (mm)	*d*_60_ (mm)	*C*_u_	*C*_c_
1.890	1.480	0.136	0.300	0.660	4.853	1.003

**Table 2:** Triaxial shear strength test scheme of EICP-cemented standard sand.

Dry Density *ρ*_d_ (g/cm^3^)	Cementation Times *n*	Standing Times *t*_1_ (d)	Confining Pressure *σ*_3_ (kPa)
1.55, 1.60, 1.65	2, 4, 6	1, 3, 5	25
1.55, 1.60, 1.65	2, 4, 6	1, 3, 5	50
1.55, 1.60, 1.65	2, 4, 6	1, 3, 5	100

**Table 3:** EICP model test scheme for erosion resistance.

Erosion Angle *α* (°)	Erosion Flow *Q* (L/min)	Erosion Times *t*_2_ (min)
15	1, 3, 5	5, 10, 15
30	1, 3, 5	5, 10, 15
45	1, 3, 5	5, 10, 15

## Data Availability

All data generated or analyzed during this study are included in this published article.
